# Influence of the Molar Mass on Long-Chain Branching of Polypropylene

**DOI:** 10.3390/polym9090442

**Published:** 2017-09-12

**Authors:** Florian Kamleitner, Bernadette Duscher, Thomas Koch, Simone Knaus, Klaus Schmid, Vasiliki-Maria Archodoulaki

**Affiliations:** 1 Institute of Material Science and Technology, TU Wien, Getreidemarkt 9, Vienna 1060, Austria; bernadette.duscher@tuwien.ac.at (B.D.); thomas.koch@tuwien.ac.at (T.K.); vasiliki-maria.archodoulaki@tuwien.ac.at (V.-M.A.); 2 Institute of Applied Synthetic Chemistry, TU Wien. Getreidemarkt 9, Vienna 1060, Austria; simone.knaus@tuwien.ac.at; 3 Pergan GmbH, Schlavenhorst 71, Bocholt 46395, Germany; dr.schmid@pergan.com

**Keywords:** long-chain branching, polypropylene, recycling

## Abstract

Long-chain branching (LCB) with peroxydicarbonates (PODIC) is known as a suitable post-reactor process to introduce strain-hardening behaviour and an increase of melt strength to a linear polypropylene (PP). This opens up new possibilities for processing and therefore application. Especially in the case of adding value to PP post-consumer waste, LCB is a promising approach. LCB takes place by a combination of chain scission and recombination after radical activation of the PP macromolecule. However, chemical modification of post-consumer waste is challenging because of the inhomogeneous composition and the manifold number of PP grades. The influence of the molar mass of the linear PP precursor on this reaction was studied with different PP grades ranging from extrusion grade to injection moulding grade. To exclude side effects, all PP grades had similar polydispersity indices. A PP with higher molar mass undergoes significant chain scission during the LCB process compared to a PP with low molar mass for injection moulding. Therefore, the two grades differ significantly in their branching number, which influences their behaviour in elongational flow.

## 1. Introduction

Isotactic polypropylene (PP) is one of the most popular consumer plastics in the global polymer market. Its applications include plastic pipes, injection-moulded building parts, chill-rolled and blow-moulded films, fibres, thermoforming, etc. Regarding other thermoplastics, PPs’ desirable properties like low density, high melting temperature, moderate stiffness and low cost make it the plastic with the second largest share in global plastic production [1,2]. Conspicuously, most applications of PP are designated only for short-term single use. As a result, a big share of the produced PP ends up quickly in post-consumer plastic waste. Establishing viable recycling processes is still a challenge as most PP post-consumer waste is incinerated or goes to landfill [3].

Mechanical recycling requires reliable analytical methods for quality assessment but would be the method of choice for the reintroduction of post-consumer waste PP in the manufacturing process of new PP products [4]. For example, the recycling of lead and PP from car batteries is a successful concept [5]. However, these processes are limited as PP is prone to photo-oxidation and degradation processes during its lifetime and mainly during the re-processing procedure due to the presence of tertiary carbons [6,7,8,9]. PP post-consumer waste collected from municipal household waste is a commingled feedstock with irregular composition. The impurities in PP post-consumer waste can be categorised into four groups: high molecular impurities like other plastics; low molecular impurities like different stabilisers; homo molecular impurities like PPs with different molar masses, or *co*-polymers; and inorganic impurities like fillers or metals [10,11]. While plastics with significant higher densities like polyethylene terephthalate (PET) and polyvinylchloride (PVC) can be separated by flotation in water, the similar densities of polyethylene (PE) and PP impede a simple separation [12]. For the reuse of PP/PE-fractions, compatibilisers like ethylene-propylene-rubber (EPR) are added to reduce negative influences from the immiscibility of PE and PP [13,14]. In the literature, concepts like in situ compatibilisation of PP/PE blends are shown to be attractive and cheap alternatives. Reactive monomers and/or peroxides are used to generate interfacial crosslinks to improve the mechanical properties of the polyolefin blends [15,16,17,18,19,20,21,22]. The kinetics of such grafting reactions is of great interest and so they have been studied intensively. A novel kinetic Monte Carlo strategy was recently presented by Hernández-Ortiz et al. to model the event history of grafting reactions [23].

**Figure 1:**
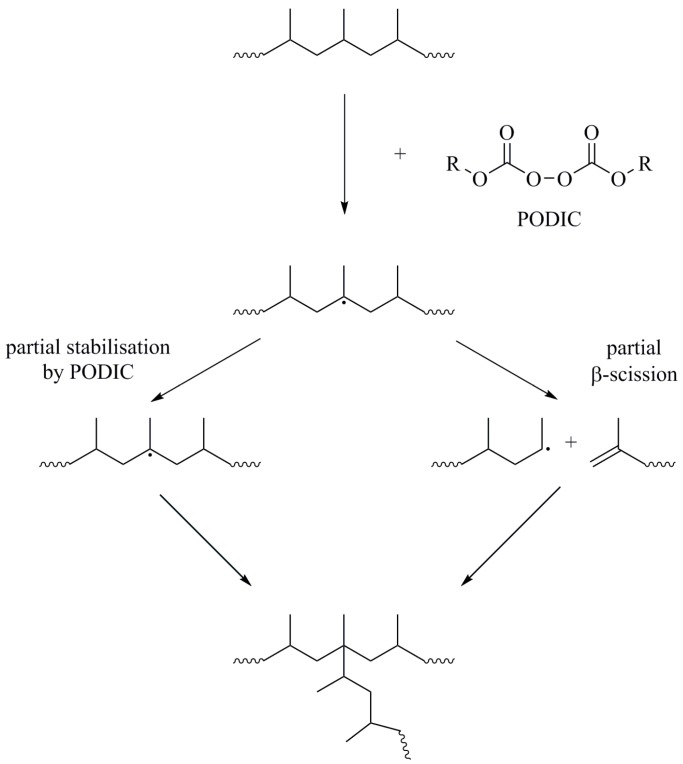
Reaction scheme of LCB process assisted by PODIC, according to Lagendijk et al.

In order to introduce long-chain branches (LCB) in linear isotactic PP, similar combinations of monomers and peroxides are used. LCB is a well-studied topic in the literature and numerous possibilities have been offered to find an efficient process to compensate for the loss of mechanical properties during recycling [24,25,26,27,28,29,30,31,32,33,34]. LCB in general results in an increase in the molar mass, a broadening of the molar mass distribution, an acceleration of crystallisation and an increase of the melt strength as well as strain hardening [34,35,36,37,38]. In case of PP, the property profile is extended due to the increased melt strength and special product applications, such as foaming and film blowing [39]. A suitable method to get LCB-PP from a linear isotactic PP homopolymer was presented by Lagendijk et al. They used peroxydicarbonates (PODIC), a special class of peroxides, to generate PP with an LCB structure [40]. PODICs with long aliphatic side chains (e.g., di-myristyl-peroxydicarbonate) are well described in a later work to give a high branching efficiency [41]. The PODIC acts as both initiator and *co*-agent and mediates partial chain scission and radical stabilisation for a successful branching reaction. A general reaction scheme is given in Figure 1.

**Figure 2:**
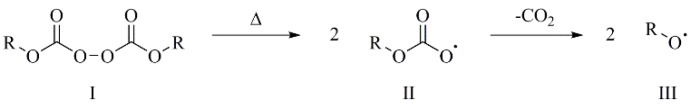
Decomposition of PODIC.

A detailed study of the PODIC-mediated LCB reaction in melt has not been provided yet. In general, two alkoxy carbonyloxyl radicals are formed by a homolytic cleavage of the O–O bond (II). In case of a sufficiently long time interval, (II) may decarboxylate by eliminating carbon dioxide, producing two alkoxy radicals (III) (Figure 2). In the case of di-myristyl-peroxydicarbonate, Buback et al. showed a high stability of species (II) [42]. Species (III) is expected to abstract hydrogen from the PP generating the PP macroradical. The improvement in melt strength is ascribed to the stabilisation of the PP macroradical due to the recombination with (II) and the formation of an alkylcarbonate-polymer adduct [43].

It could be shown that LCB is a suitable concept to improve the melt properties of PP from post-consumer waste with PE impurities, which is why one can speak of a real upcycling process [44]. The method of using PODIC and reactive extrusion is of special interest as it may be applied directly by the plastic manufacturer on recycled linear PP. However, LCB as an innovative recycling process is more promising when used for the modification of single polymer waste, because impurities like PEHD in PP post-consumer waste limit the number of possible applications of the upcycled product [45]. Nevertheless, even recycled single-polymer waste is a commingled resource of inhomogeneous composition. On the one hand, thermally and mechanically degraded PP resulting from reprocessing procedures has a high MFI, which influences the mechanical and flow properties [46,47]. On the other hand, single PP post-consumer waste contains different PP grades from different applications and therefore PPs with different molar masses. The molar mass is an important factor for the physical and mechanical properties of a polymer. For semi-crystalline thermoplastics, a higher molar mass induces an increasing number of inter- or intramolecular entanglements, a higher number of secondary bonds per volume, and changes of near- and long-range order. This directly influences the ability to crystallise, the elastic modulus and stiffness above glass transition temperature, as well as the flow properties in melt [48]. Therefore, the influence of PP grades of different molar mass on the LCB reaction and the number of LCB according to Lagendijk et al. in relation to the molar mass will be the topic of this study. In order to exclude any confounding effect of polydispersity, samples with similar *M*_w_*/M*_n_ ratios were used.

## 2. Experimental Section

### 2.1. Materials

Four different isotactic PP homopolymers supplied by Borealis (Vienna, Austria) were used for the study. PP 1 (HA 104E, Borealis, Vienna, Austria) is a high molar mass extrusion grades for pipe systems, PP2 (HC 600TF) is intended for thermoforming applications, PP3 (HD 601CF, Borealis, Vienna, Austria) is a film resin for chill roll processes, and PP4 (HF 700SA, Borealis, Vienna, Austria) is a PP grade for injection moulding. The different PPs differ only in their molar mass (*M*_w_ and *M*_n_), but have similar dispersities (*M*_w_*/M*_n_). Their rheological and molecular data are summarised in Table 1.

PODIC (a white powder) Peroxan C126 (Pergan, Schlavenhorst, Germany) (Di-tetradecylperoxydicarbonate; 10 h half-life at 48 °C) was supplied by Pergan (Schlavenhorst, Germany).

### 2.2. Thermal Analysis

A standard procedure with TA Instruments (TA) standard aluminium pans (5 mg sample mass) on a TA Q2000 DSC (TA instruments, Newcastle, DE, USA) was used for thermal analysis. Samples were heated to 200°C (10 °C·min^−1^), cooled down to room temperature and heated up again to 200 °C at the same rate. TA Universal analysis software (TA instruments, Newcastle, DE, USA) was used to determine melting (*T*_m_) and crystallisation temperature (*T*_c_), as well as the melting enthalpy (*ΔH*_m_). 

Oxidation induction time (OIT) was determined according to DIN ISO 11357-6 [49]. The samples were heated up in open pans to 200 °C (10 °C·min^−1^) under a nitrogen atmosphere. Then the purging gas was changed to air and the temperature was kept at 200 °C for 90 min.

### 2.3. Molar Mass Determination

The determination of the molar mass distribution (MMD) was carried out on a Viscotek High Temperature size exclusion chromatography (HT-SEC) system (Malvern instruments, Herrenberg, Germany) at 140 °C with 1,2,4-Trichlorobenzene as eluent and standard triple detection (refractive index, low angle light scattering and capillary viscometer).

### 2.4. Rheology

Discs with 25 mm diameter and 1.2 mm thickness for dynamic rheology were compression moulded at 20 bar and 180 °C. Dynamic rheology measurements were carried out on a plate-plate Anton Paar MCR 301 rheometer (Anton Paar, Graz, Austria) equipped with a CTD 450 heating chamber (Anton Paar, Graz, Austria) under nitrogen at 180 °C with 1 mm gap size. The frequency range was set from 628 to 0.01 rad·s^−1^ and deformation was raised logarithmically from 1% to 2% during the measurement. All measurements were performed within the linear viscoelastic region and thermal stability was checked by time sweep experiments at 180 °C for 2 h. The zero-shear viscosity η_0_ of the samples was determined by measuring the creep compliance at 180 °C at a constant stress τ of 5 and 30 Pa. For small stresses, there is a linear range, whereby the creep compliance *J*(*t,*τ) reaches a stationary state and the zero-shear viscosity can be determined from the plateau of *t/J* when it is plotted against *t*.

(1) $$\eta_{0}=\underset{t\to \infty }{\lim}\left(\frac{t}{J\left(t,\tau \right)}\right)$$

**Figure 3:**
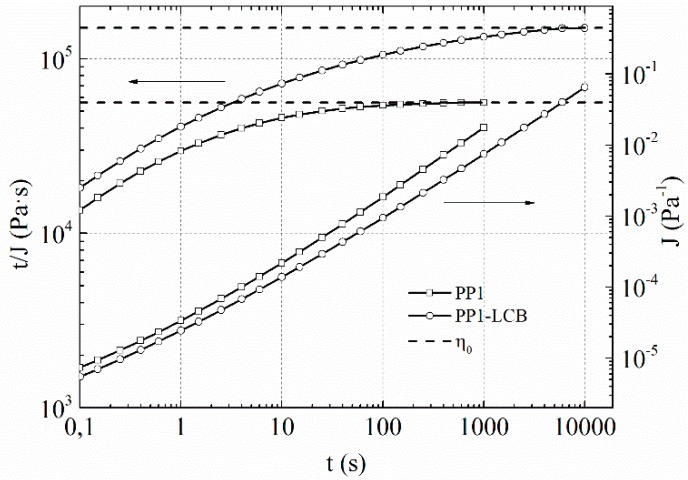
Creep experiments and determination of η_0_.

This is shown in Figure 3 for PP1 and PP1-LCB.

### 2.5. Extensional Rheology

Stripes with 8 mm width for extensional rheology were cut from 100 mm × 100 mm × 0.5 mm sheets after compression moulding at 180 °C. Extensional rheology was measured using a Sentmanat Extensional Rheometer (SER-HPV 1, Xpansion instruments, Tallmadge, OH, USA) for Anton Paar rheometers, at 180 °C and five different strain rates ($\dot{\varepsilon }$ = 10; 3; 1; 0.3; 0.1 s^−1^). The strain hardening ratio SHR was calculated using the maximum value of the elongational viscosity $\eta_{\mathrm{Emax}}^{+}$ for every strain rate and the corresponding value at time *t* from the threefold of the linear viscoelastic start up curve.

(2) $$SHR=\frac{\eta_{\mathrm{Emax}}^{+}\left(t,\dot{\varepsilon }\right)}{3\eta^{+}\left(t\right)}$$

### 2.6. Sample Preparation

**Figure 4:**
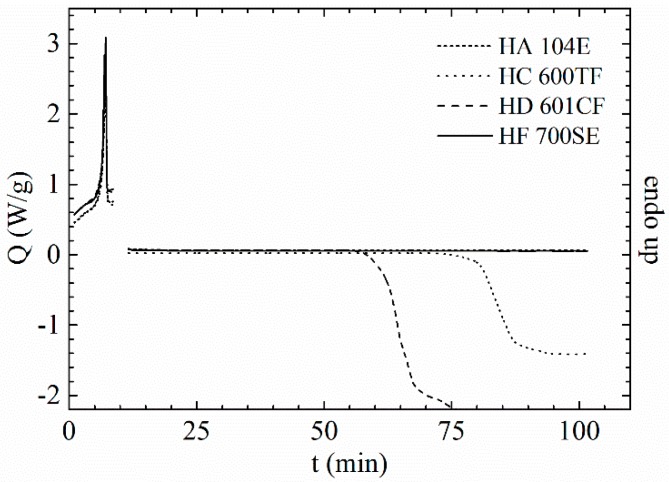
Comparison of the OIT of the shredded PP granules.

Particles with a mean diameter of 1 mm were formed by shredding virgin PP granules with a Fritsch granulator “Pulverisette 16, Fritsch, Idar-Oberstein, Germany”. From the literature it is known that stabiliser systems (especially hindered amine light stabilisers and sulphur-containing additives) can influence the melt modification of PP [28,50]. Therefore, the oxidation induction time (OIT) of the polypropylene grades was determined to compare the oxidative behaviour of the materials. PP1 for pipes and PP4 for injection moulding had an OIT longer than 90 min (Figure 4). To minimise possible side effects, the stabilisers were washed out of the grinded polymers PP1, PP2 and PP3 by Soxhlet extraction overnight with acetone. In the case of PP4, it was necessary to repeat the procedure with dichloromethane. The OIT of the industrial grade and after Soxhlet extraction are summarised in Table 2.

A solution of the PODIC in 50 ml *n*-hexane was purified on the destabilised grinded polymer, mixed and then stored at room temperature until the solvent evaporated. Reactive extrusion was carried out in a Haake Mini Lab II conically shaped twin-screw extruder at 180 °C and with 100 rpm screw speed for 5 min. After 4 min, when the PODIC should be completely decomposed (estimated from the half-life), 1 mg (about 0.02%) Irganox 1010 (BASF, Ludwigshafen, Germany) was added in order to prevent further degradation.

## 3. Results and Discussion

### 3.1. Thermal Analysis

**Figure 5:**
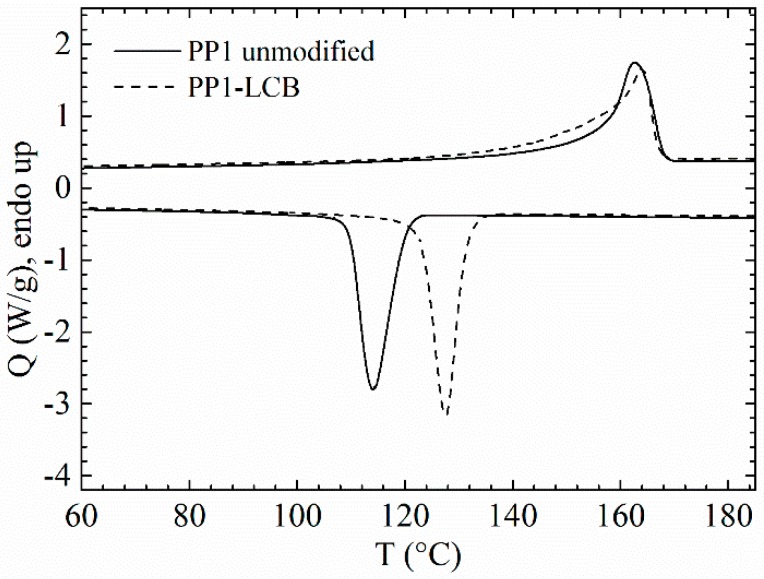
Changes in thermal properties for linear and LCB-PP1.

The degree of crystallinity of semi-crystalline polymers depends—besides the processing conditions—on the molar mass and the structural regularity of a given polymer. It can be reflected by the enthalpy of fusion *ΔH*_m_, and the melting temperature *T*_m_ and is reduced by the introduction of non-crystallising structural units such as branches and grafts [48]. During the radical induced melt modification of PP, degradation and recombination take place simultaneously, which results in a higher *M*_w_, a broadening of MMD and more chain irregularity. Therewith the crystallisation behaviour of the LCB-PP is affected. Wang et al. described the crystallisation behaviour of LCB-PP prepared by grafting of pentaerythritol triacrylate (PETA). They suggested that the small number of branching points in LCB-PP increases the nucleation density, which results in higher *T*_c_ [51]. Compared to its linear precursor, Tian et al. calculated smaller Avrami exponents for LCB-PP, which was also prepared by grafting with PETA. They concluded that LCB acts as a heterogeneous nucleating agent and influences the mechanism and the growth of PP crystals [52]. Also, Nam et al. suggested irregularities induced by long-chain branching to broaden the melting peak of the LCB-PP [39]. Tabatabei et al. studied blends of linear and LCB-PP, and postulated that even a small amount of LCB increases the number of nuclei sites, resulting in an increase of crystallinity. However, an increased number of branches prevents chain mobility, leading to a decrease in crystallinity (20% of LCB-PP in the blend) [53]. Nevertheless, *ΔH*_m_, *T*_m_ and *T*_c_ increased for all samples (shown in Figure 5). 

The extent of the increase of *ΔH*_m_, decreases from PP1-LCB over PP2-LCB and PP3-LCB to PP4-LCB and therefore so does the effect of PODIC modification on the crystallisation behaviour.

As can be seen in Table 3, the extent of the increase of *ΔH*_m_ decreases from PP1-LCB over PP2-LCB and PP3-LCB to PP4-LCB. Therefore, the effect of PODIC modification on the crystallisation behaviour is more pronounced in a high molar mass grade PP compared to the injection-moulding grade, with an innately high ability to crystallise.

Further conclusions relating changes of the polymer structure (e.g., an average number of branches per molecule) cannot be drawn from the DSC data, but it is a fast and reliable method and gives a hint of the success of the LCB reaction.

### 3.2. MMD and LCB of the PP Samples

**Figure 6:**
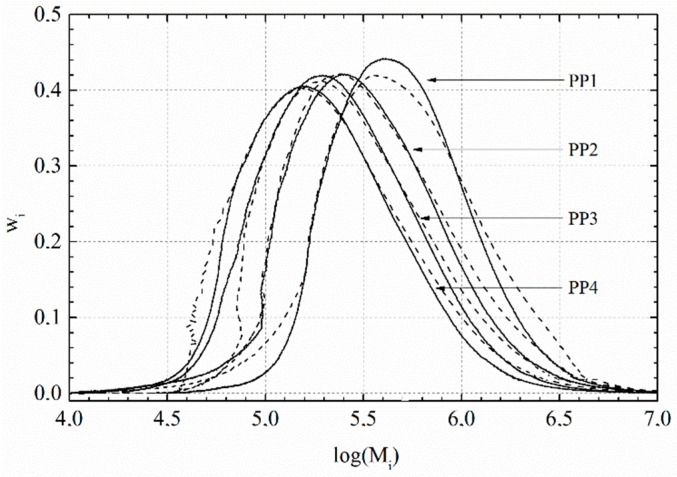
MMD of the linear PP samples (compact lines) and the LCB samples (dashed lines).

The weight average molar mass, the number average molar mass, and the polydispersity indices and the number of branches per molecule are summarised in Table 4. The MMD of all polymer samples is shown in Figure 6. The LCB samples (dashed lines) show a distinctive shoulder in the high molar mass region. This shoulder looks more pronounced when the linear PP has a higher molar mass. *M*_w_ and *M*_w_/*M*_n_ thus increased for all samples. Additionally, the peak maximum of the MMD of PP1-LCB was distinctively reduced and the peak also broadens in the region of smaller molar masses.

**Figure 7:**
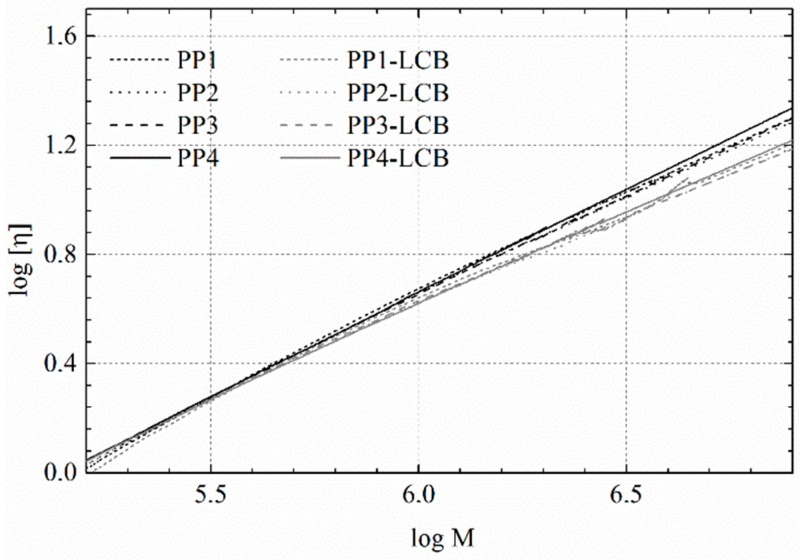
Intrinsic viscosity as a function of the molar mass of the linear PPs (black lines) and the LCB PPs (grey).

The branched structure of the LCB-samples can be detected from the Mark–Houwink plot in Figure 7, by comparing the solution of the LCB-sample with its linear precursor under the condition of a similar molar mass. Comparison with a linear equivalent should also enable the quantification as well as the detection of branching. In Table 4, the average number of branches per molecule *B*_n_ was calculated according to the model of Zimm and Stockmeyer [54,55], using the method of Lecacheux et al. [56]. This method has a number of limitations, but allows for an accurate quantification of branching, e.g., for PE [57,58], and is therefore also commonly used for PP.

(3) $$g={\left(\frac{{\left[\eta \right]}_{b}}{{\left[\eta \right]}_{l}}\right)}^{\frac{1}{\epsilon }}$$

The ratio of the mean square radii of gyration *g* was calculated from the intrinsic viscosity of the branched [η]_b_ and the linear [η]_l_ polymer. The parameter ϵ depends on the type of branched structure and the solvent–polymer interaction. In the literature, ϵ has a value of 0.5 for star polymers, 1.5 for combs with large backbones and short branches, and 0.7 for multi-arm stars [59,60,61]. For LCB-PP a value of ϵ = 0.75 is often used in the literature [40,62,63]. *B*_n_ was calculated by solving the following equation and the results are summarised in Table 4:

(4) $$g={\left[{\left(1+\frac{B_{n}}{7}\right)}^{\frac{1}{2}}+\frac{4B_{n}}{9\pi }\right]}^{-\frac{1}{2}}.$$

The results in Table 4 show that *B*_n_ increases, with a decrease of the molar mass of the linear PP. According to the reaction scheme in Figure 1, PP1 (with high molar mass) forms larger chain fragments with reduced ability for recombination during LCB due to lower chain mobility, which is in accordance with [64]. This results in a decreasing branching number *B*_n_ and longer side arms with a higher molar mass. The prevalence of LCB (and increasing *B*_n_) seems enhanced, with a lower molar mass of the linear unmodified PP.

### 3.3. Dynamic Rheology

**Figure 8:**
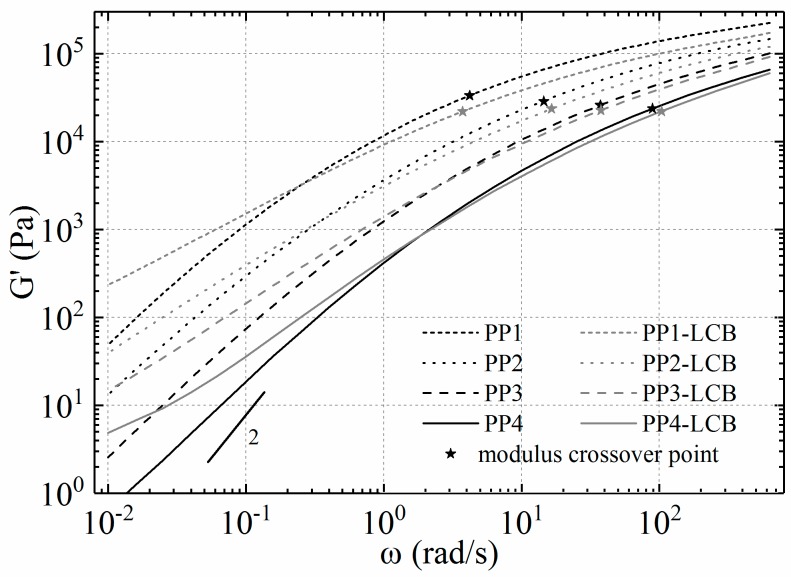
Storage moduli as a function of angular frequency at 180 °C.

The linear viscoelastic behaviour of a polymer melt is sensitive to structural changes of macromolecules, especially to LCB. Therefore, the comparison of dynamic moduli is a well-established technique to show the structural changes of a polymer. LCB primarily influences the elastic behaviour and therefore the storage modulus of the polymer samples in the low-frequency regime. The storage moduli of linear precursors and the LCB-PPs are presented in Figure 8. According to the predictions of Fleissner et al., changes in *M*_w_ and MMD (e.g., induced by the LCB) influence the position of the modulus crossover point [65]. The crossover modulus of all samples decreases with LCB (broadening of MMD) and the crossover frequency shifts towards smaller values (higher *M*_w_). Except for PP1, its crossover frequency shifts towards a slightly higher value (smaller *M*_w_), which is in accordance with the data from SEC. 

**Figure 9:**
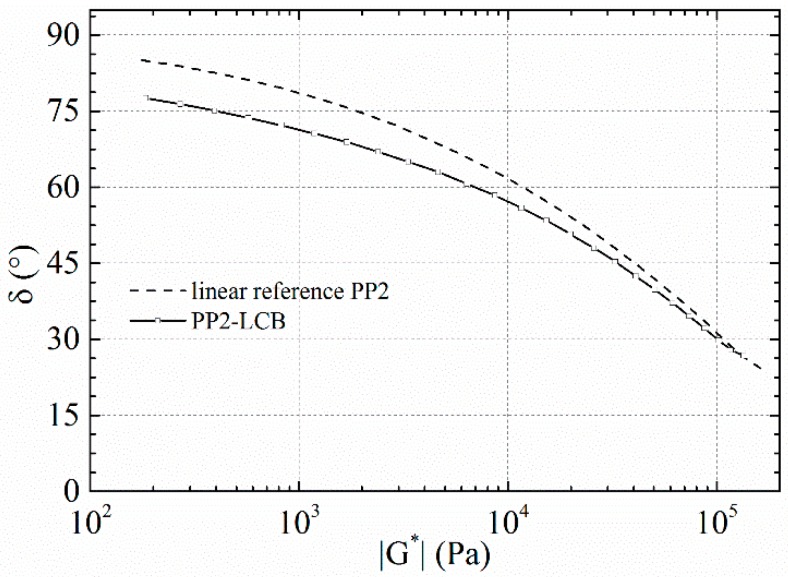
δ-|G*|-plot of PP2 (linear reference) and PP2-LCB.

Additionally, the storage modulus of PP4-LCB shows a clear and PP3-LCB a slight deviation from the slope of 2 in the low frequency range, which may include a second elastic plateau. According to Wood-Adams [61], such a second plateau is a further hint of the branched structure of the measured polymer. A plot of the loss angle δ and the complex modulus |G^*^| (van Gurp Palmen plot) offers the possibility to classify and quantify the amount and type of LCB with a linear reference of similar MMD [66,67]. The deviation from the linear reference depends strongly on the degree of LCB (which is shown in Figure 9 for PP2 and PP2-LCB). In our case a lightly LCB structure can be concluded from the plot.

**Figure 10:**
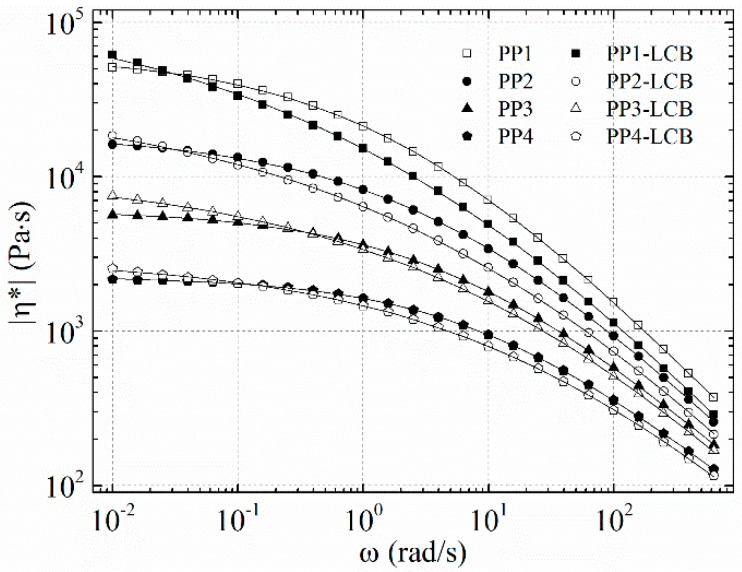
Dynamic viscosity as a function of angular frequency at 180 °C and the prediction of the Carreau–Yasuda model.

Further characteristic effects of a branched structure, when compared to a linear precursor, are a pronounced shear thinning at high shear and a higher viscosity at low shear. As can be seen in Figure 10, the linear PPs reach their zero-shear viscosity plateau between the frequency of 0.1 rad·s^−1^ and 1 rad∙s^−1^, while the transition zone from power law to the zero-shear regime shifts to smaller frequencies and becomes broader for the LCB-PPs. The effect of shear thinning is more pronounced with PP1-LCB and decreases with diminishing molar mass.

According to Tsenoglu et al., the branching number *B*_n_ can also be calculated from the measured zero-shear viscosity of the branched PP η_b_ and the linear PP η_l_, especially for LCB-PP prepared from a linear PP with PODIC [68]. The theory assumes that for sparsely branched polymers the number of branch points per molecule is either zero or one, and the melt is approximated to a blend of mostly linear with three-arm star-shaped chains with arm length half of the average length of the linear precursor. Under these assumptions, the fraction of branched molecules equals *B*_n_, which can be calculated with Equation (5):

(5) $$B_{n}=\frac{\ln\left\{\frac{\eta_{b}}{\eta_{l}}\right\}}{\alpha \left[\frac{M_{L}}{M_{C}}-1\right]-3\ln\left\{\frac{M_{L}}{M_{C}}\right\}}.$$

In this equation *M*_L_ is the weight average molar mass of the linear PP, *M_C_* the molecular weight at the onset of entanglements, which is equal twice of the molecular weight of two successive entanglements (*M*_C_ ≈ 2*M*_e_)*,* and the numerical coefficient α = 0.42. According to the tube model, α = 15/8, but experiments indicated a lower value of *α* ≈ 0.43–0.60 for star-shaped polymers [68]. In a later work, Gotsis et al. used a modified α = 0.48 for a LCB-PP with lower molar mass compared to their previous work and postulated that α is dependent on the molar mass and the molar mass distribution [35]. For PP1-LCB and PP2-LCB results of *B*_n_ were obtained with α = 0.42 similar to *B*_n_ from HT-SEC. For PP3-LCB and PP4-LCB α = 0.42 gave no satisfying results. Jørgensen et al., for example, needed to adapt α = 0.8 to get the best fit for their LCB-metallocene HDPE [69]. We also used adapted α values to get the best fit for PP3-LCB and PP4-LCB (results are shown in Table 5). However, the values are within the experimental values of α ≈ 0.43–0.60, which are given in the literature.

### 3.4. Extensional Rheology

**Figure 11:**
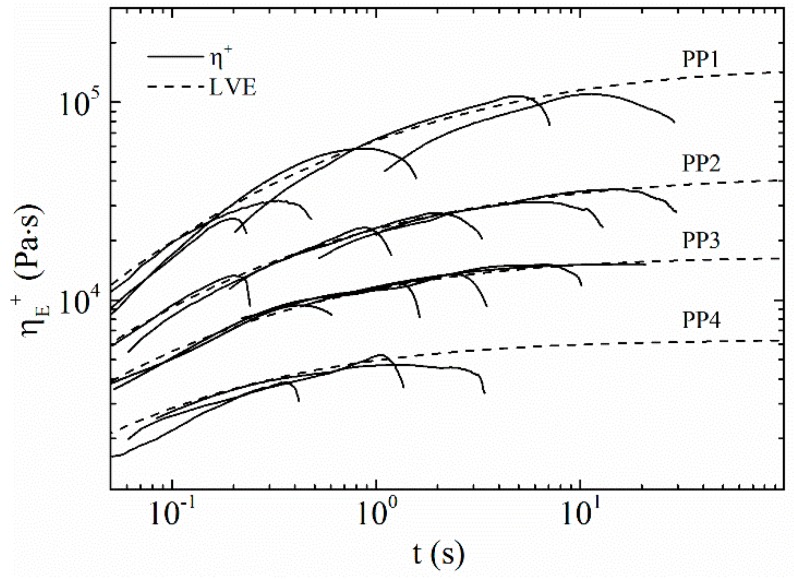
Extensional rheology of the linear PPs.

Distinct differences in the elongational behaviour are related to changes in the molecular structure of the studied PP. LCB-PPs are able to build up a strong entangled network connected with strong nonlinear effects of the elongational viscosity called strain hardening. As can be seen in Figure 11, the linear PPs show no strain-hardening behaviour and no deviation from the linear viscoelastic start-up curve. Because of the low zero-shear viscosity of PP4 at the measuring temperature, it was not possible to measure the extensional viscosity at Hencky strain rates below 1 s^−1^.

**Figure 12:**
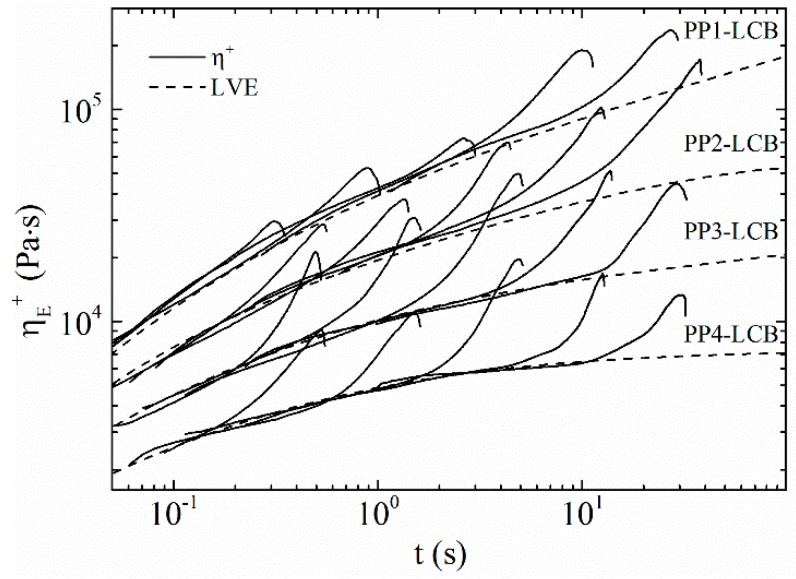
Extensional rheology of the LCB-PPs.

**Figure 13:**
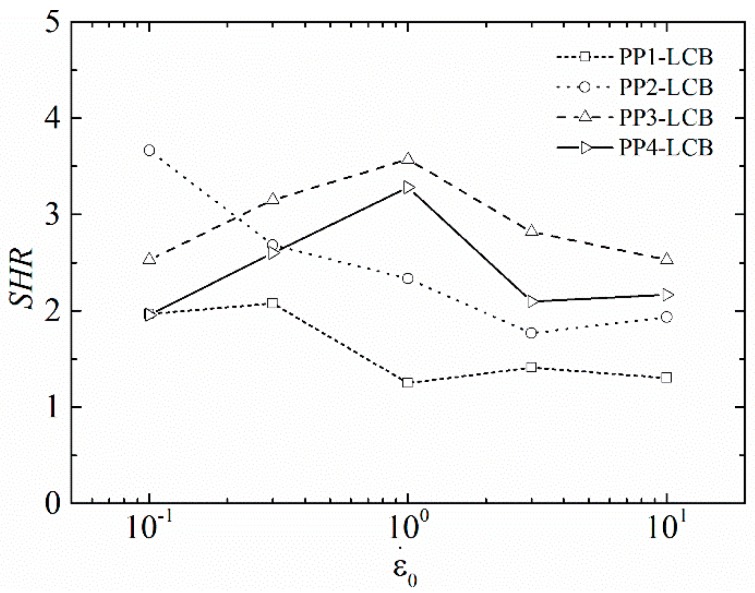
Strain hardening ratio of the LCB samples.

The modification with PODIC induced LCB to the linear polymer backbone and therefore strain-hardening behaviour was obtained for all LCB-PPs. (It has to be noted that PP3-LCB and PP4-LCB show slight sagging, which is, especially at lower strain rates, in competition with the strain hardening.) The SER curves are plotted in Figure 12 and the calculated corresponding strain-hardening ratios are given in Figure 13. The SHR increases with increasing *B*_n_, which can be seen in Figure 13 when the SHR at ε = 1 s^−1^ of PP1-LCB, PP2-LCB and PP3-LCB are compared. This is in agreement with the results from HT-SEC and the calculated *B*_n_. All samples show a dependency of the strain hardening on the strain rate. The SHR of PP1-LCB shows a slight decrease of SHR with increasing strain rate; this becomes clearer for PP3-LCB. The SHR of PP3-LCB and PP4-LCB first increase and reach their maximum at ε = 1 s^−1^ and then decrease again. According to Gabriel et al. [70], more pronounced strain hardening at the lower Hencky strain rate is a sign of fewer branches in the polymer, which is in agreement with the calculated *B*_n_. However, PP3-LCB and PP4-LCB do not exactly follow the observations from Gabriel at al. but lie between the predictions. This seems obvious because the *B*_n_ for PP3-LCB and PP4-LCB are not enough so speak from highly branched.

## 4. Conclusions

In this study a peroxydicarbonate (PODIC) with long aliphatic side chains was used for the LCB of different PP grades. The linear PPs differed in their average molar mass but had similar polydispersity indices. LCB-PPs were prepared via reactive extrusion in melt. The melt viscosity of the LCB-PPs showed classical effects such as an increase in melt elasticity and pronounced strain hardening, which is in agreement with the literature. Furthermore, strain-hardening behaviour was introduced to all LCB-PPs. A strain-hardening dependency of all samples was observed. PP1-LCB and PP2-LCB showed more pronounced strain hardening at low strain rates, which is typical of a low number of branches. LCB was proven by HT-SEC measurements and the deviation of the intrinsic viscosity of the branched samples from their linear PPs was shown. The average branching number per molecule was calculated according to the model of Zimm and Stockmeyer and showed a very low value for PP1-LCB (pipe grade) of 0.08; this value increases to 0.13 for PP2-LCB (from thermoforming grade), and to 0.25 and 0.27 for PP3-LCB (from casting grade) and PP4-LCB (injection moulding grade), respectively. The number average molar mass of PP1-LCB and PP2-LCB decreased compared to their linear feedstocks. Due to the radical reaction mechanism of the LCB, it is suggested that the recombination reaction and therefore the resulting LCB is inhibited by the higher molar mass of PP1. In contrast, the lower molar mass fragments of PP4 lead to a higher number of LCBs. These results will have to be considered for the upcycling of commingled PP single-polymer waste and will be the basis of further studies.

## Figures and Tables

**Table 1:** Rheological and molecular data of the PPs used as raw materials for the study.

Sample	MFI ^a^ [g/10 min]	η_0_ [Pa·s]	*M*_w_ [kg mol^−1^]	*M*_n_ [kg mol^−1^]	*M*_w_*/M*_n_
PP1	0.75	56 600	559	321	1.74
PP2	2.8	17 700	394	198	1.99
PP3	8	5 900	300	151	1.99
PP4	21	2 140	227	99	2.29

^a^ provided in the product data sheet.

**Table 2:** OIT before and after Soxhlet extraction of the PPs.

Sample	OIT	OIT
	Industrial Grade	after Soxhlet
	[min]	[min]
PP1	>90	17
PP2	67	9
PP3	48	10
PP4	>90	11

**Table 3:** Thermal properties of the studied polymer samples.

Sample	*T*_m_ [°C]	*T*_c_ [°C]	*ΔH*_m_ [J·g^−1^]
PP 1	163	114	90
PP 1-LCB	164	128	98
PP 2	161	113	92
PP 2-LCB	163	128	98
PP 3	162	115	93
PP 3-LCB	163	128	99
PP 4	162	114	98
PP 4-LCB	163	127	101

**Table 4:** Molar masses, polydispersity and branching number of the LCB-PPs.

Sample	*M*_w_ [kg mol^−1^]	*M*_n_ [kg mol^−1^]	*M*_w_*/M*_n_	*B*_n_
PP1-LCB	611	317	1.92	0.08
PP2-LCB	436	196	2.22	0.13
PP3-LCB	333	151	2.20	0.25
PP4-LCB	264	106	2.49	0.27

**Table 5:** Branching number from rheological data.

Sample	η*^0^* [Pa·s]	*B*_n_	α
PP1-LCB	150 200	0.08	0.45
PP2-LCB	37 100	0.13	0.46
PP3-LCB	11 500	0.25	0.49
PP4-LCB	2730	0.27	0.52
